# Study on biodegradation of Mazut by newly isolated strain Enterobacter cloacae BBRC10061: improving and kinetic investigation

**DOI:** 10.1186/1735-2746-10-2

**Published:** 2013-01-02

**Authors:** Alireza Chackoshian Khorasani, Mansour Mashreghi, Soheyla Yaghmaei

**Affiliations:** 1 Department of Chemical and Petroleum Engineering, Sharif University of Technology, Tehran, Iran; 2 Cell and Molecular Research Group, Institute of Biotechnology, Ferdowsi University of Mashhad, Mashhad, Iran

**Keywords:** Biodegradation, Mazut, Gradual addition, Biokinetic model, *Enterobacter cloacae*

## Abstract

Mazut as a source content of various hydrocarbons is hard to be degraded and its cracking could turn mazut into useful materials. Nevertheless degradation of mazut by routine methods is too expensive but application of indigenous microorganisms as biocatalysts could be effective and important to lower the costs and expand its consumption. Mazut biodegradation can be improved using various strategies; Therefore in this study newly isolated strain *Enterobacter cloacae* BBRC 10061 was used in a method of gradual addition of mazut into medium and its results were compared with simple addition method. To investigate degradation of mazut by BBRC 10061, influence of increase of mazut concentration was assayed based on gradual addition method. Also different kinetic models were used to evaluate kinetics of the process. Results showed that gradual addition method has been a beneficial technique for improvement of mazut degradation because bacterial induction to produce biosurfactant and essential enzymes for cracking mazut was higher during process. Although addition of more mazut increased the rate of biodegradation but percentage of degradation decreased. pH of medium decreased during biodegradation period while electric potential increased. Also the biodegradation kinetics was not fitted with the biokinetic models; therefore kinetics of biodegradation of mazut has to be studied by new models.

## Introduction

End product of crude oil distillation is a residual compound named mazut that the presence of heavy hydrocarbon compounds such as asphaltene, resins, long chain alkanes, cyclic alkanes and multi cyclic aromatics in it turn mazut into a high viscous and hard degradable product; On the other hand, sulphuric compounds and heavy metals in mazut make use of it more difficult. Mazut has various types based on hydrocarbon compounds which usually depended on geographical area and storage sources which crude oil is prepared from [1]. Bioprocess is suggested as effective and economical provident ways. Its advantage against other physical and chemical processes is simplicity and lower cost [2-5]. What makes biodegradation as a most important process is use of newly isolated indigenous microorganisms [6]. Biodegradation of oil derivatives contaminating environments is more effective, powerful and economical provident than physical and chemical ways [7]. Assessment of effective parameters and conditions on biodegradation is very significant to design bioremediation system [8]. Due to material and process, microorganism can be isolated from environments which their conditions prepare microorganism to turn material on demand. Isolating from indigenous area is usually advantageous to achieve high microbial performance; moreover preparation of appropriate conditions for microorganism can help procedure because microorganism is adapted by them [9]. Various studies have already been reported about detection and isolation of able microorganisms to degrade oil compounds but there are not enough reports of biodegradation of mazut by microorganisms in Iran. Indigenous microorganisms such as *Pseudomonas aeruginosa, Pseudomonas stutzeri, Provedenica stuartii, Acinetobacter* sp, *Bacillus Cereus, Micrococcus* sp, *Enterobacter cloacae* and *Serratia* sp isolated from different locations in Iran which were contaminated by oil compounds, such as soil and water sources, were employed to degrade various hydrocarbons but not mazut [10-15]. Biokinetic models are mathematical tools to predict and model activity and behavior in a biosystem so that can investigate different kinetic parameters and rate curves based on real and controlled data [16,17]. Therefore enough knowledge of microbial growth and degradation kinetics of matters are necessary to model biokinetics [18]. Each of simple biokinetic models already been suggested is usually adapted to special conditions and suitable to particular processes. Among models, Monod model (1949) is simplest model already been reported and other models such as Moser model (1958), Webb model (1963), Aiba et al. model (1968), Haldane model (1968), Yano and Koga (1969), Teissier model (1970) and Levenspiel model (1980) describing kinetic behavior of complex biosystems are advanced [19,20].

Objectives of this study were investigation of effect of gradual addition of mazut on biodegradation of hydrocarbon derivative in mazut using an indigenous bacterium *Enterobacter cloacae* BBRC 10061 isolated from oil contaminated soil in Mashhad (Iran), and modulation of its biodegradation kinetics.

## Materials and methods

### Microorganism

*E. cloacae* BBRC 10061, named NO4 in this study, is indigenous bacteria strain isolated from oil contaminated soil where buses were fixed and fuelled in Mashhad, Iran. It was procured from the biochemical and bioenvironmental research center (BBRC) that was a local culture collection in Sharif University of Technology; Tehran, Iran. The microorganism was maintained in glycerol stock at −20°C for further use.

### Culture medium and experimental condition

The medium culture had the following composition per liter: MgSO_4_.7H_2_O 0.1 g, CaCl_2_ 0.01 g, FeSO_4_.7H_2_O 0.01, (NH_4_)_2_SO_4_ 5 g, KH_2_PO_4_ 2.5 g, K_2_HPO_4_ 2.5 g, sea salt 0.5 g, glucose 4 g and tween80 1 g. The medium was autoclaved at 121°C for 20 min and after cooling, sterile medium was distributed in 50 ml sterile centrifuge tubes which were inoculated with NO4 bacteria amounted to 0.032 OD_600_.

Two types of culture were used, simple and gradual. To prepare simple culture, 2000 ppm (0.1 g per 50 mL) mazut was added into 50 ml medium culture in centrifuge tube. Gradual culture was prepared at concentrations of 2000 ppm, 10000 ppm and 50000 ppm of mazut. Every 2 day, 400 ppm, 2000 ppm and 10000 ppm mazut were added in centrifuge tubes containing 50 ml medium cultures to prepare gradual cultures in concentrations of 2000 ppm, 10000 ppm and 50000 ppm.

All cultivations were then incubated for 10 days at 33°C and initial pH 6.8 with a speed of 160 rpm.

### Analytical methods

For estimation of mazut concentration, spectrophotometer (S2000 UV/VIS) was used. To extract mazut from medium, chloroform was added into medium proportionately with initial concentration of mazut (3 ml chloroform for < 2000 ppm, 6 mL for < 10000 ppm and 9 mL for < 50000 ppm) and centrifuge tube was shaken and blended till all mazut was dissolved in chloroform. Then organic phase content of mazut and chloroform was under and mineral phase was up. To extract organic phase, mineral part voided of mazut and another organic components dissolved in chloroform was decanted. After that, organic phase was sampled and diluted by chloroform solvent. Now absorbance of mazut in medium was measured at 450 nm by spectrophotometer. Sample concentration was measured based on absorbance-concentration curve.

To estimate bacteria amount in medium, after sampling from medium bacterial density was measured at 600 nm by spectrophotometer and recorded as optical density (OD_600_).

Electric potential and pH were measured by pH meter (3020 model, Jenway).

### Biokinetic models

Results of degraded mazut in this study were fitted on various biokinetic models which are presented in Table 1 where r_s_ is rate of mazut degradation (mg/ L /day), r_s max_ is maximum of mazut degradation rate (mg/ L/ day), S is mazut concentration (ppm), x is microbial optical density (dimensionless), K_s_ is half-saturation constant of mazut (mg/ L), K_i_ is inhibition constant (mg/ L), K_1_ is inhibition constant (mg/ L), K_2_ is inhibition constant (mg/ L), λ is constant of Moser model (dimensionless), β is constant of Contois model (mg/ L), A (mg/ L /day), B (day), C (mg /L/ day) and D (mg/ L /day) are constants of linear models. Experimental data for the degraded mazut and the bacteria were used as the experimental data to validate the models. The mathematical models were solved numerically in Microsoft Office Excel 2007 using the SOLVER tool. The kinetic parameters and constants for each timepoint were calculated based on the mazut concentration. A linear regression between the experimental data and the predicted data were computed for all the assays.

**Table 1:** Biokinetic models used for biodegradation of mazut

**Model**	**Equation**
Monod	$r_{s}=r_{s\max}\frac{S}{K_{s}=S}$
Teissier	$r_{s}=r_{s\max}\left(e^{-\frac{S}{K_{i}}}-e^{-\frac{S}{K_{s}}}\right)$
Aiba et al.	$r_{s}=r_{s\max}\frac{S\left(e^{-\frac{S}{K_{i}}}\right)}{K_{s}+S}$
Yano and Koga	$r_{s}=r_{s\max}\frac{\text{S}}{K_{s}+S+\frac{S^{2}}{K_{1}}+\frac{S^{3}}{K_{2}^{2}}}$
Haldane (Andrews)	$r_{s}=r_{s\max}\frac{\text{S}}{K_{s}+S+\frac{S^{2}}{K_{1}}}$
Webb	$r_{s}=r_{s\max}\frac{S\left(1+\frac{\text{S}}{\mathrm{Ki}}\right)}{K_{s}+S+\frac{S^{2}}{K_{1}}}$
Levenspiel	$r_{s}=r_{s\max}\frac{S\left(1+\frac{\text{S}}{\mathrm{Ki}}\right)}{K_{s}+S}$
Moser	$r_{s}=r_{s\max}\frac{1}{1+K_{s}\left(S^{-\lambda }\right)}$
Contois	$r_{s}=r_{s\max}\frac{\text{S}}{\mathrm{\beta x}+S}$
First order	r_s_ = aS + b
Second order	r_s_ = aS^2^ + bS + c
Third order	r_s_ = aS^2^ + bS^2^ + cS + d

## Results

### Influence of gradual addition of mazut

**Figure 1:**
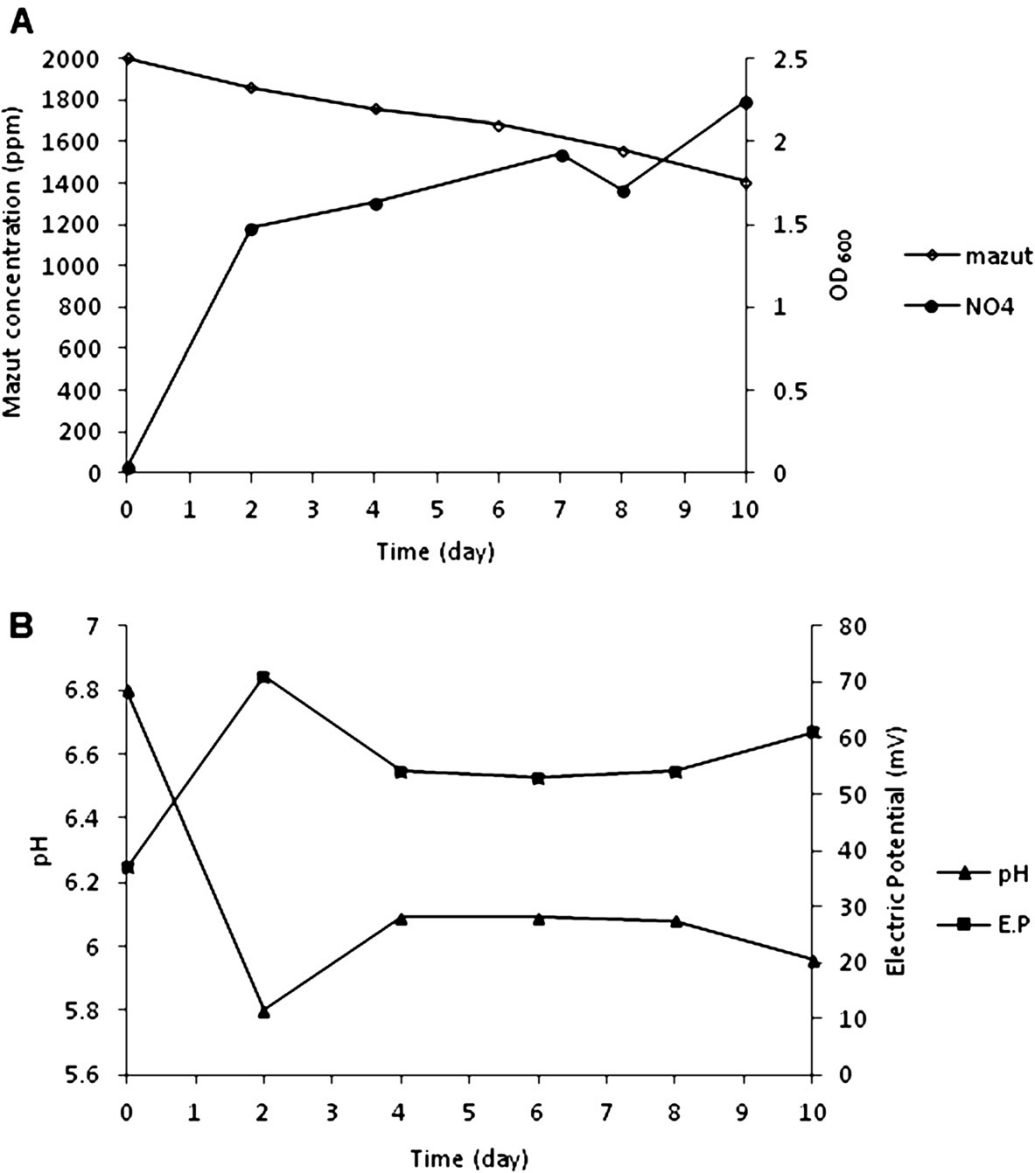
(A): Biodegradation of mazut and bacterial growth in simple medium (B) pH and electric potential change during mazut biodegradation in simple medium.

**Figure 2:**
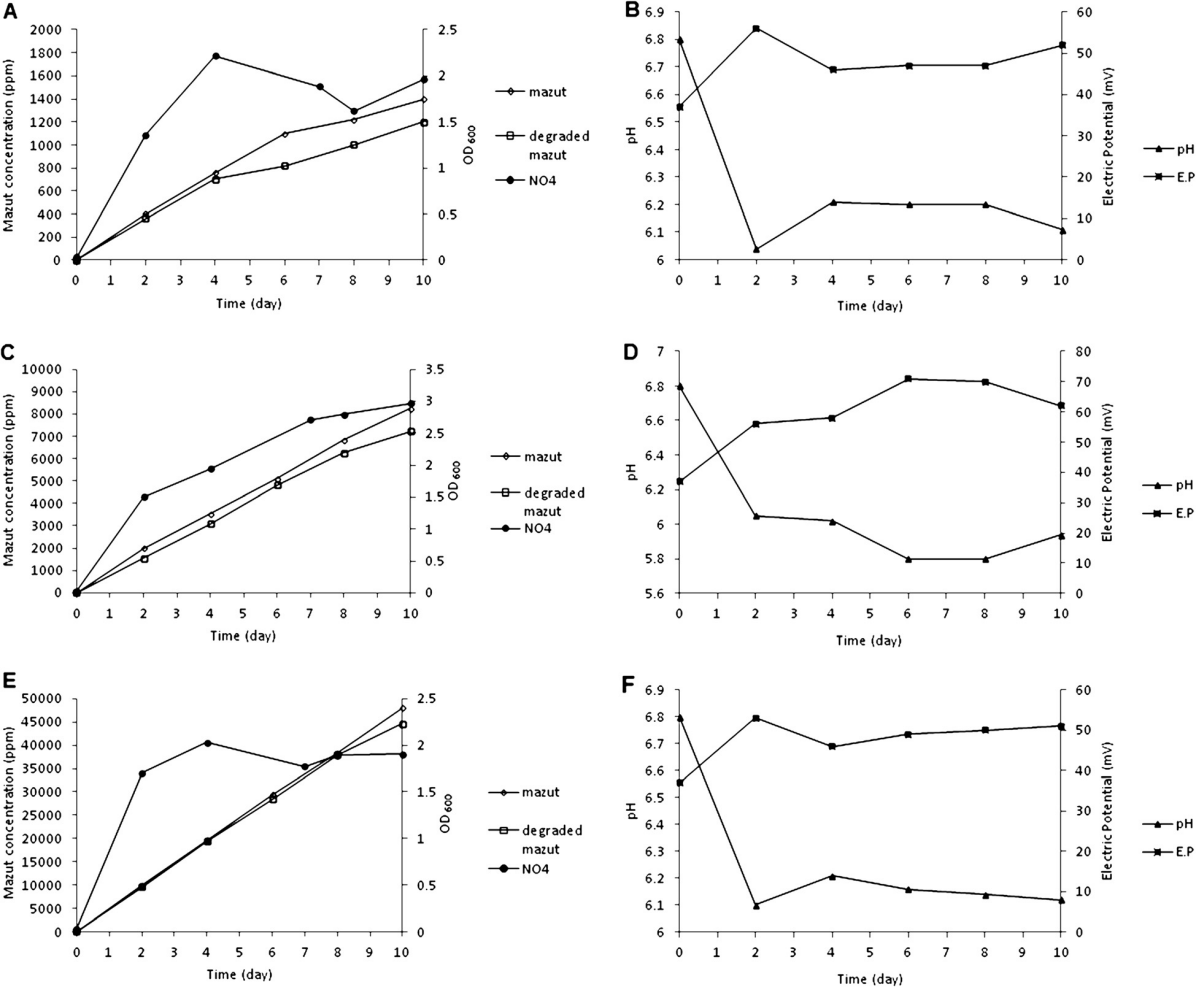
**(A):Biodegradation of mazut and bacterial growth in gradual medium at 400 ppm additional concentration.** (**B**) pH and electric potential change during mazut biodegradation in gradual medium at 400 ppm additional concentration. (**C**) Biodegradation of mazut and bacterial growth in gradual medium at 2000 ppm additional concentration. (**D**) pH and electric potential change during mazut biodegradation in gradual medium at 2000 ppm additional concentration. (**E**) Biodegradation of mazut and bacterial growth in gradual medium at 10000 ppm additional concentration. (**F**) pH and electric potential change during mazut biodegradation in gradual medium at 10000 ppm additional concentration.

Degradation commenced after adding 2000 ppm mazut into medium and during the process NO4 bacteria degraded mazut about 1400 ppm (Figure 1A). Biodegradation rate was steady but bacterial growth rate fluctuated. Bacterial growth increased quickly at process beginning and with time lapse rate decreased although bacteria did not enter into death phase. According to Figure 2A efficiency of degradation of mazut was very low until fourth day but after that degradation rate became steady. During ten days from 2000 ppm mazut added into medium just 1200 ppm remained indicating to about %40 mazut degradation. NO4 bacterial growth at first four days attained the most amounts and after then bacteria entered into stationary phase. Growth rate increased with degradation of simple hydrocarbons but reduced after deployment and degradation rate remained unchanged because cracked hydrocarbons turned into more digestible derivatives and bacteria grew by consuming them whereas main molecules of mazut were degraded at foregoing rate and it was approximately independent of biomass change. Gradual addition caused biosystem to be slowly adapted and this method in general could be more effective than abrupt addition at start; because abrupt addition of mazut may cause nutrimental shock and microbial dead resulting decrease of growth rate. Bacterial growth was very fast in first 2 days while the rate of mazut degradation was very low (Figure 2A). These changes showed stage of adaptivity of microorganism to culture medium. Bacteria grew by consuming glucose in medium and were ready to degrade mazut. Then bacterial growth did not change and mazut was degraded in stationary phase. This status was better than status which mazut degradation occurred during microbial growth because during process, amount of biomass as catalyst was approximately fixed constant. Also this amount was equivalent to final amount of biomass at simple method. Thus it caused degradation percentage became higher in longer period of time with more microorganisms presented. Bacterial growth was accompanied by secondary acidy products and medium turned to acidy quickly by increase of microbial growth rate in first days but at following process pH change was very low. The reason was that acidy condition decreased bacterial activity and produce of secondary products. Complications of production and consumption of different materials in system caused many changes in electric potential and conductivity in culture medium which were difficulty legitimized; but totally during the biodegradation of mazut pH reduced and electric potential increased in medium.

### Influence of mazut concentration

For assessment of effectiveness of gradual method, influence of increase in mazut concentration on biodegradation rate was studied by this method. With increasing additional concentration of about 2000 ppm in which during ten days 10000 ppm was in medium, mazut degradation percentage attained to %30. At this time residual mazut got 7000 ppm and biomass with less fluctuated growth gained about 3 OD_600_ (Figure 2C). Gradual addition for every stage was 2000 ppm but degradation time of 2 days was very short. Only 3000 ppm was degraded at ten days and bacterial growth was slow. Bacterial growth and mazut degradation were possible during 10 days due to mazut remained at every stage and NO4 bacteria did not enter into stationary phase. Increase in concentration of mazut caused inducing bacteria to degrade it and finally more concentration was degraded. Percentage of mazut degradation by NO4 bacteria attained to %10 when concentration of mazut in biodegradation system increased to 50000 ppm (Figure 2E). Maximum bacterial growth was observed at first 2 days and with entering into the stationary phase biodegradation process was carried out that final biomass gained about 2 OD_600_. In first days of incubation, pH reduced from 6.8 to 6 relating to increase in microbial growth which made medium acidy (Figure 2D, F). Electric potential increased at the beginning of incubation periods but when microbial growth approached stationary phase, changes in pH and electric potential was not observed. The reason for pH reduction or increase in electrical conductivity of medium could be due to increase of production and consumption of different materials which are the results of increase in biomass and rate of degradation.

### Evaluation of biokinetic models

Biodegradation of mazut at different concentrations using simple and gradual medium was fitted on the biokinetic models which their results are presented in Table 2. The results showed that simple process did not correlate with any models but gradual process at 2000 ppm additional concentration correlated with second and third order models. Other gradual processes data were not fitted on the models. Results also showed that given biokinetic models were more suitable to model gradual processes. Although in general mazut biodegradation data was not proper to be modeled by given models but second and third order models were more correlated than other models.

**Table 2:** Linear correlation coefficients for simple and gradual mediums

**Biokinetic Model**	**Simple medium**	**Gradual medium (400 ppm additional concentration)**	**Gradual medium (2000 ppm additional concentration)**	**Gradual medium (10000 ppm additional concentration)**
	**R^2^**	**R^2^**	**R^2^**	**R^2^**
Monod	0.07	0.57	0.52	0.56
Teissier	0.06	0.57	0.52	0.56
Aiba et al.	0.16	0.57	0.52	0.56
Yano and Koga	0.1	0.60	0.52	0.56
Haldane (Andrews)	0.16	0.57	0.52	0.56
Webb	0.12	0.63	0.50	0.63
Levenspiel	0.07	0.57	0.52	0.56
Moser	0.08	0.56	0.50	0.57
Contois	0.00	0.02	0.35	0.56
First order	0.07	0.57	0.52	0.56
Second order	0.85	0.80	0.98	0.77
Third order	0.88	0.85	0.95	0.68

## Discussion

There are just a few studies on biodegradation of mazut compared with other materials such as crude oil and other petroleum compounds. Therefore this project has been a new research and investigation in the field of petroleum biotechnology. New isolated strain of bacterium *E. cloacae* in this study was capable to biodegrade mazut efficiently and was of first bacterial strain cracking mazut. More researches on mazut biodegradation have already been carried out in soil medium whereas this study was completely investigated in controlled specific water condition. Bioremediation of mazut-polluted soil by fungi species reduced 300 mg of initial concentration which was 5000 mg per kg soil during ten days and further incubation up to140 days reduced mazut concentration down to 1500 mg per kg soil [21]. Among fungus studied, species *Trichoderma harzianum* had highest ability for degradation of mazut [22]. Results obtained from biodegradation of mazut in soil using ex situ method showed that during 50 days 3 g of 5 g mazut in 1 kg soil was degraded and finally after 150 days got decreased into 0.5 g per kg soil concentration [23]. Using gradual addition of mazut method caused quick growth and adaptation of bacteria at initial days. Thus biodegradation of mazut carried out by most possible amount of bacteria nevertheless in simple method amount of bacteria became maximal at the end of process, and due to that gradual addition increased biodegradation of mazut to %10 more. Increase of mazut concentration resulted increase in efficiency of degraded mazut; nevertheless percentage of degradation decreased. The changes due to increasing concentration were not linear and whatever percentage of increase of mazut was more, percentage of degradation fell down progressively since at very high concentrations increase of concentration had no effects on degradation.

Changes in pH of medium were appropriate for bacterial growth and activity. Simultaneously with higher rate of microbial growth, reduction in value of pH was faster and medium became more acidified. This phenomenon acted as an inhibitor factor for bacterial growth and caused stopping growth. After fast growth of bacteria at starting days, no growth was observed when pH unchanged and became constant. In this study, most amount of reduction of pH was about 1 unit in all of the assays. During the biodegradation of mazut, materials in medium were chemically changed as conductivity of medium was affected by it. Biodegradation of mazut resulted increase of medium’s electric potential that was maximum at growth time. Usually in degradation of hydrocarbons, increase of microbial activities can cause quick reduction of pH which is a reason for reduction of microbial growth; as at following process pH was constant [24-27]. Electric potential is a parameter which assigns amount of ions. Changing in ions amount directly relates to change in electric potential in each medium. Microbial activities alternate ions amount but changes are different in relation to each ion in a medium. Basically, ionic changes in bacterial activities are affected with hydrogen ions, because changes in other ions, which are consumed as nutrients or are produced, are not sensible. Thus, decrease of pH, increasing hydrogen ions, practically raises electric potential. In this study, pH changes directly affected on electric potential. Whatever rate of pH changes became higher, rate of electric potential changes got higher. The recorded data demonstrated close relevance between pH and electric potential changes. Consequently, measuring electric potential in medium can help estimating pH without measuring it. On the other hand, comparing pH trend with electric potential trend can indicate sensible presence of other ionic materials during microbial activities.

Increase of mazut concentration induced bacteria to consume more mazut. Results showed that addition of mazut into medium decreased percentage of biodegradation but more amount of mazut was degraded. Addition of every concentration of mazut could increase degradation but only up to certain amount in which increase in concentration would not raise degradation capacity. After 21 days degradation of heavy crude oil by *E. cloacae* at 50000 ppm, 10000 ppm and 2500 ppm concentrations were %10, %40 and %53 respectively. At high concentrations of heavy crude oil degradation percentage did not change and increasing concentration decreased microbial growth and degradation percentage [11]. Usually in biodegradation of hydrocarbons, increase of hydrocarbon concentration raised degraded hydrocarbon and reduced percentage of degradation [24,28-31]; nevertheless increase of concentration was able to decrease [24,25] or increase [31] microbial growth.

In general, comparing with other studies we could able more clearly show the effective mazut degradation by gradual additional method because there is not any report published about kinetics of biodegradation of mazut. This study could be first in this type of investigation and more studies can be carried out in future. The biokinetic models could be most powerful models, although were not so much correlated with biodegradation of mazut; hence, there is demand for new more powerful model to predict mazut biodegradation process.

## Conclusion

Mazut were efficiently degraded by newly isolated bacteria. Mazut biodegradation was improved with gradually addition of substrate. Substrate addition allowed bacteria to gradually adapt to a heavy hydrocarbon medium. Gradually adaptation raised biodegradation rate, increasing biomass. Increasing mazut concentration reduced degradation percentage because bacterial growth and consequently, degrading enzymes production were limited in a batch medium. Changing in pH and electric potential of medium were fluctuated during biodegradation. In the early day, decrease of pH and increase of electric potential were occurred; then, their oscillations were reduced. Electric potential change could be evidence for pH alternation, which can help to tune pH in the medium without measuring pH. Investigation of mathematical models on mazut biodegradation stated complexity of mazut biodegradation to mathematically modeling. Therefore, modeling mazut degradation, and using substrate addition technique can be studied to improve other biodegradation processes.

## Competing interests

The authors declare that they have no competing interests.

## Authors’ contributions

ACK, carried out this study as part of his M.Sc. project, planed the original research of the project, and wrote the manuscript. MM, supervised the microbial part of this research, and revised the manuscript. SY, supervised the mathematical part of the project, and read the manuscript. All authors read and approved the final manuscript.
